# Engineered microvascular basement membrane mimetic for real‐time neutrophil tracking in the microvascular wall

**DOI:** 10.1002/btm2.70008

**Published:** 2025-03-12

**Authors:** Laura C. Morales, Catherine D. Kim, Yangang Pan, Simon Scheuring, Anjelica L. Gonzalez

**Affiliations:** ^1^ Department of Biomedical Engineering Yale University New Haven Connecticut USA; ^2^ Department of Anesthesiology Weill Cornell Medical College New York New York USA; ^3^ Department of Physiology & Biophysics Weill Cornell Medical College New York New York USA; ^4^ Kavli Institute at Cornell for Nanoscale Science Cornell University Ithaca New York USA

**Keywords:** basement membrane, microvasculature, neutrophil, pericyte, real‐time microscopy

## Abstract

The microvascular basement membrane (mvBM) is crucial in maintaining vascular integrity and function and, therefore, key to the health of major organs. However, the complex nature and the intricate interplay of biochemical and biomechanical factors that regulate the mvBM functional dynamics make it difficult to study. Here, we present a novel and highly tunable in vitro model of the human mvBM, enabling a bottom‐up approach to assemble a composite model of the microvascular wall and explore microvascular dynamics and interactions with circulating neutrophils in real time. An electrospun polyethylene glycol (PEG)‐based fibrillar network mimics the mvBM with adjustable nanofiber diameter, orientation, and density. The fidelity of the model to the human mvBM's topography and mechanics was verified through second harmonic generation imaging and atomic force microscopy. PEG was functionalized with bioactive moieties to enable endothelial cell (EC) and pericyte (PC) attachment, through which neutrophil interactions with the microvascular wall model were observed. The model, coupled with 4D microscopy, revealed nuanced and dynamic neutrophil behavior when interacting with the microvascular wall, demonstrating its utility in characterizing cell–cell interactions. As such, the model can be employed in the exploration of inflammatory and microvascular‐related diseases. Therefore, this innovative approach represents a significant advancement in vascular biology research, holding profound implications for understanding mvBM dynamics in both health and disease.

**Translational Impact Statement**

Understanding microvascular‐related diseases require novel therapeutic strategies. Developing new therapies necessitates in vitro models of microvascular microenvironments that emulate physiologically relevant conditions and offer real‐time monitoring of cellular dynamics. Here, we present a highly tunable in vitro mimetic of the human microvascular basement membrane that enables a detailed study of microvascular dynamics, including vascular wall remodeling and cell trafficking, both crucial for understanding the mechanisms underlying these conditions.

**Abbreviations**
AFM: atomic force microscopy
BM: basement membrane
EC: endothelial cell
ECM: extracellular matrix
HUVEC: human umbilical vein endothelial cells
ICAM‐1: intercellular adhesion molecule 1
mvBM: microvascular basement membrane
NG2: neuroglial antigen 2
PC: pericyte
PDGFR‐β: platelet‐derived growth factor‐β
PECAM‐1: platelet endothelial cell adhesion molecule 1
PEG: polyethylene glycol
PEGDA: polyethylene glycol diacrylate
PEO: polyethylene oxide
RGDS: arg‐gly‐asp‐ser
SA: surface area
SAV: surface‐area‐to‐volume
SEM: scanning electron microscopy
SHG: second harmonic generating
TNF‐α: tumor necrosis factor‐α
αSMA: α smooth muscle actin

## 1 INTRODUCTION

The basement membrane (BM) is a submicron‐scale collagen IV and laminin network, interconnected by additional proteins such as perlecan and nidogen.^1^, ^2^, ^3^, ^4^ These proteins and others form an intricately organized and specialized extracellular matrix (ECM). Despite the fundamental composition of BMs being relatively consistent across different tissues, the complexity of individual BMs are highly dependent on the specific cell types they surround or support.^5^, ^6^, ^7^ In the microvasculature, the BM is situated between the luminal endothelial layer and the pericytes (PCs) that line the outer circumference of the vessel. Endothelial cells (ECs) and PCs actively contribute to the deposition and organization of this specialized microvascular ECM structure, which, in turn, regulates cellular processes, including adhesion, migration, proliferation, and differentiation.^1^, ^6^, ^8^, ^9^ Moreover, the microvascular BM (mvBM) acts as a physical barrier to regulate the passage of cells across the vascular wall, which occurs during the inflammatory transmigration of immune cells from the bloodstream into the tissues.^9^, ^10^, ^11^

Far from a static substrate, the mvBM displays dynamic characteristics, continuously undergoing changes through protein synthesis, degradation, and reorganization.^12^, ^13^ These dynamic modifications profoundly influence the composition of the mvBM, as well as its topography and mechanical properties, intricately shaping the behavior of vascular cells. Dysregulated changes to the mvBM can instigate and advance various pathological conditions, including diabetes,^14^ fibrosis,^12^, ^15^ and dermatosis,^16^ in which mvBM remodeling leads to aberrant neutrophil recruitment. Therefore, understanding the precise role of the BM in health and disease becomes paramount, especially when developing targeted therapies to improve patient outcomes in inflammatory conditions.

Despite recent advances, the mvBM remains largely understudied, likely due to its intrinsic complexity and difficulty in exploring how singular and synergistic biochemical and biomechanical factors contribute to function.^17^ Models of the mvBM have been developed to better understand the properties of this specialized ECM.^5^, ^18^, ^19^, ^20^, ^21^, ^22^ The inherent complexity of the microenvironment found in in vivo models often hinders access to the mvBM and the examination of individual drivers of function. Conversely, in vitro mvBM models offer the advantage of creating tunable models to decouple biochemical and biomechanical alterations of the mvBM. To date, however, in vitro models of the mvBM have often focused on a single element or overlooked critical components of the vascular wall, such as PCs, and the microenvironment's role in modulating vascular cell behavior, thereby limiting their applicability. Consequently, there is an ongoing need to develop innovative and controlled in vitro mimetics of the human mvBM with physiologically relevant and tunable properties that permit real‐time monitoring of cellular activity.^11^ This is particularly crucial for investigating the rapid and dynamic fluctuations in cell state and behavior that occur during acute or pathological inflammatory neutrophil recruitment.

This study presents a novel approach to address the existing limitations of in vitro mvBM‐mimetics. We report the development of a highly tunable submicron‐level mesh scaffold that resembles the biochemical and biophysical properties of the mvBM. This engineered model was fabricated using electrospinning to create PEG fibrillar networks with adjustable parameters such as nanofiber diameter, density, and alignment. Recognizing the critical need for further exploration of the biophysical properties of the mvBM,^11^, ^23^, ^24^ we employed the use of second harmonic generation (SHG) imaging and atomic force microscopy (AFM) to characterize the human microvascular BM's morphology and mechanical properties, which served as a guide for the design constraints of our in vitro system. The fibrillar scaffold was rendered bioactive by incorporating the ubiquitously adhesive peptide arginine‐glycine‐aspartate‐serine (RGDS), which enabled the culture of human microvascular EC and PC, either separately or in a bilayer, to create a construct of the microvascular wall. This construct supported matrix remodeling events as evidenced by the deposition of collagen IV and laminin by both vascular cell types. Moreover, we integrated our mvBM mimetic with an in‐house, customized imaging chamber to evaluate the interactions between migrating neutrophils and the vascular wall components using 4D microscopy. This allowed us to observe real‐time interactions between neutrophils and each component of the microvascular wall. This versatile and highly tunable mvBM‐mimetic scaffold holds significant promise for advancing our understanding of the dynamic cellular behaviors and interactions within the vascular microenvironment. Importantly, this model can be applied to study various vascular‐related diseases. It has the potential to lead to the development of novel therapeutic strategies by facilitating the identification of key drivers of disease and providing a robust preclinical platform to test the new treatments.

## 2 MATERIALS AND METHODS

### 2.1 PEGDA synthesis

Polyethylene glycol diacrylate (PEGDA) with a molecular weight of 35 kDa was synthesized using a protocol adapted from previous studies.^18^, ^25^ Polyethylene glycol (PEG; Sigma‐Aldrich, St. Louis, MO, USA) was dissolved in anhydrous dichloromethane (American Bioanalytical, Natick, MA, USA). This solution was then reacted with acryloyl chloride (Sigma‐Aldrich) and triethylamine (Sigma‐Aldrich) for 24 h while maintained under argon. Induction of phase separation between the organic and aqueous components was achieved through the addition of potassium carbonate (Sigma‐Aldrich). The organic phase was collected, and the reaction was quenched using magnesium sulfate (JT Baker, Center Valley, PA, USA). The resulting product was precipitated in ice‐cold diethyl ether (Sigma‐Aldrich), followed by dialysis and lyophilization for further purification of the unreacted product.

### 2.2 Nanofibrillar scaffold fabrication

Polyethylene oxide (PEO) with a molecular weight of 400 kDa obtained from Sigma‐Aldrich was dissolved in double‐distilled water to form a 3.2% (w/v) solution. PEO served as a carrier polymer, facilitating the formation of fibers through polymer chain–chain entanglement. To enhance conductivity, sodium chloride (Sigma‐Aldrich) was added to the PEO solution at a concentration of 1% (w/v). PEGDA was dissolved in the PEO solution at concentrations ranging from 20% to 30% (w/v). Pentaerythritol triacrylate, obtained from Sigma‐Aldrich, was added as a crosslinker for PEO at a concentration of 8% (v/v), and N‐vinyl pyrrolidone (300 mg/mL in acetophenone) served as a photoinitiator at a concentration of 10 μL/mL.

**FIGURE 1:**
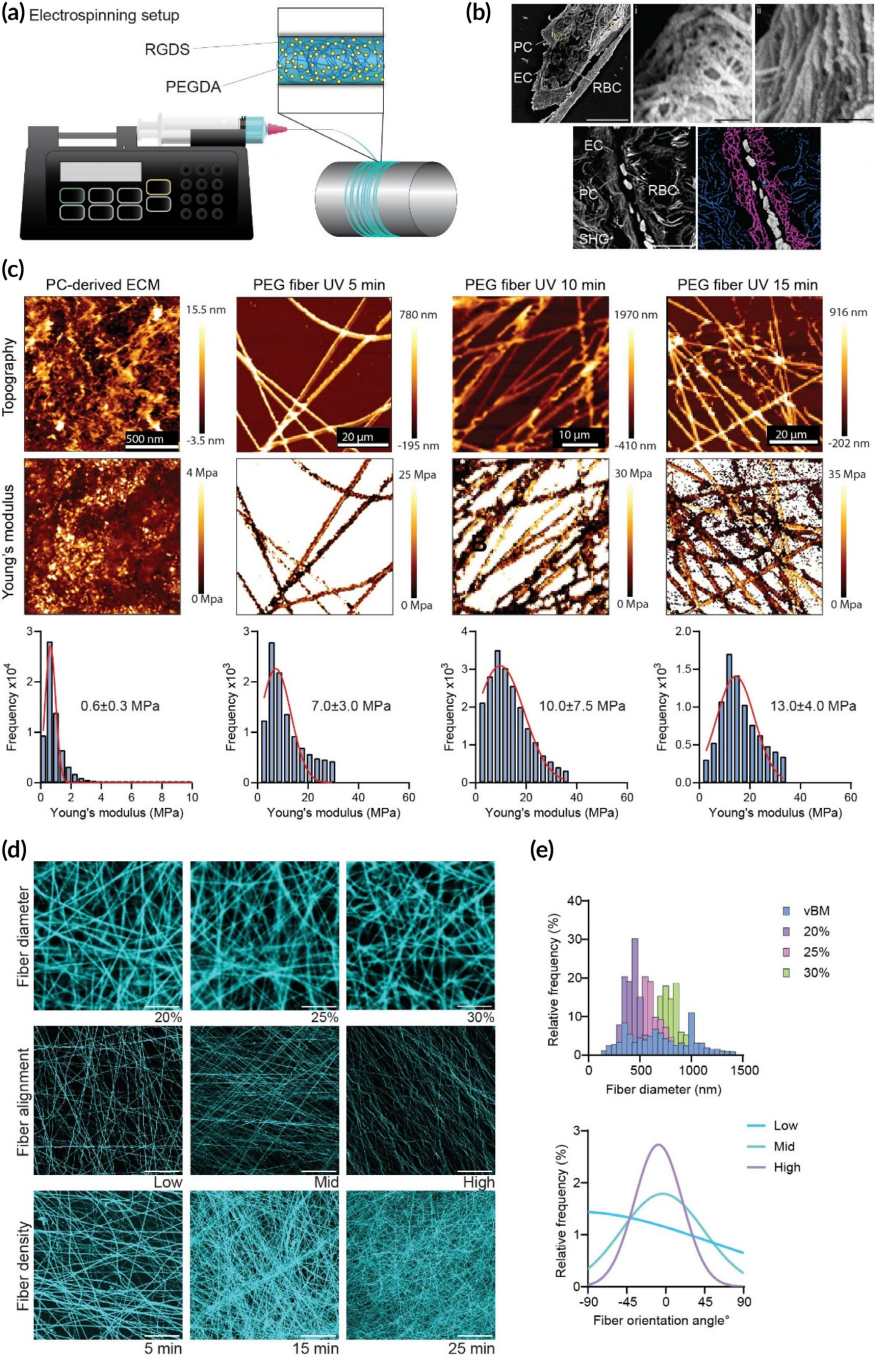
Characterization of electrospun fibrillar matrices with adjustable mechanical and morphological features. (a) Schematic illustration of the electrospinning apparatus detailing fiber composition. (b) SEM and SHG images of human placental microvessels. Scale bars from left to right, top down: 20 μm, 200 nm, 200 nm, 20 μm. (c) Representative AFM image of de novo synthesized PC ECM and electrospun PEG meshes subjected to UV exposure for 5, 10, or 15 min. Quantification of AFM data for calculation of Young's moduli of PC‐BM and fibrillar matrices crosslinked for varying times. (d) Confocal microscopy images displaying rhodamine‐labeled PEG fibers at various diameters, fiber alignments, and densities. Scale bar diameter: 10 μm. Scale bar alignment and density images: 50 μm. (e) Frequency distributions of fiber diameter for mvBM and 20%, 25%, and 30% PEGDA fibers. Analysis of fiber orientation under low, medium, and high collection speeds, accompanied by frequency distributions of fiber orientation angles.

The synthesis of nanofibrillar networks was carried out through electrospinning. The electrospinning setup included a high‐voltage power supply (Glassman High Voltage Inc., High Bridge, NJ, USA), a syringe pump (Harvard Apparatus, Holliston, MA, USA), and a grounded metallic collecting drum. PEO‐PEGDA solutions were electrospun using a 26G needle (Hamilton, Reno, NV, USA) at a flow rate of 2 μL/min, an applied voltage of 15 kV, and a tip‐to‐collector distance of 20 cm (Figure 1a). Fiber orientation was controlled via collecting drum speed variations. The electrospun fibrillar mats were crosslinked by subjecting the membranes to ultraviolet (UV) light for varying durations, enabling precise control over the degree of crosslinking and stiffness of the resulting matrix.

### 2.3 Acrylate‐PEG‐RGD synthesis

To render the scaffolds bioactive for cell studies, the peptide arginine‐glycine‐aspartate‐serine (RGDS) obtained from Lifetein (Somerset, NJ, USA) was presented on the surface of the PEG nanofibers. The functionalization of the nanofibrillar surface involved coating the fibers with acrylate‐PEG‐RGDS (Ac‐PEG‐RGDS). The synthesis of Ac‐PEG‐RGDS was accomplished by conjugating RGDS to the available succinimidyl carboxymethyl ester (SCM) groups in Ac‐PEG‐SCM sourced from Creative PEGWorks (Durham, NC, USA). The reaction was conducted in a 50 mM sodium bicarbonate buffer at pH 8.5, with a 1:1 molar ratio of reactants. To conjugate RGDS groups onto the fibrillar scaffold, a solution containing 10% Ac‐PEG‐RGDS and 10 μL/mL of crosslinker was applied to the membranes and allowed to soak for 3 min. The membranes were then exposed to UV light for 1 min and washed in 1X phosphate‐buffered saline (PBS) for 24 h prior to cell seeding.

### 2.4 Neutrophil isolation

Neutrophils were isolated from healthy human donors in accordance with the Yale Internal Review Board protocol 0902004786, as described in Lauridsen et al.^19^ In brief, 10 cc of blood was drawn into a syringe containing citrate phosphate dextrose (Sigma‐Aldrich). The whole blood was then transferred into a 15 mL conical, and neutrophils were isolated by immunomagnetic depletion using the MACSxpress® Whole Blood Neutrophil Isolation Kit (Miltenyi Biotech, Bergisch Gladbach, Germany), following the manufacturer's recommendations. Neutrophils were resuspended to a final concentration of 10^6^ cells/mL in Mg^+2^ and Ca^+2^ PBS and glucose and labeled with CellTracker™ Green (Thermo Fisher Scientific, Waltham, MA, USA) according to the manufacturer recommendation.

### 2.5 Microvessel isolation

Human placental microvessel fragments were isolated using a previously established protocol.^26^ Placental microvessels were used for both isolation of human pericytes for culture and imaging of mvBM (Figure 1b). Briefly, a section of a human placenta was dissected under sterile conditions and rinsed with PBS. The tissue was then finely minced using surgical scissors. This was followed by enzymatic digestion using Collagenase Type D (Sigma‐Aldrich) to facilitate the dissociation of microvessels from the surrounding tissue matrix. The digested tissue suspension underwent double filtration, enabling the effective separation and subsequent collection of placental microvessel fragments.

### 2.6 Cell culture and substrate seeding

Human umbilical vein endothelial cells (HUVECs) were harvested following the procedures described in Gimbrone et al.^27^ and cultured in 0.1% gelatin‐coated tissue culture flasks. Briefly, HUVECs isolated from a collagenase treatment of term umbilical cord veins were cultured in EGM™‐2 Endothelial Cell Growth Basal Medium (Lonza, Basel, Switzerland), supplemented with the EGMTM‐2 Endothelial SingleQuotsTM Kit (Lonza). All HUVECs (ECs) were used between passages 2 and 4. Human placental PCs were isolated from microvessels obtained from donated anonymized human placentas according to the methods described in Maier et al^26^ PCs were cultured in tissue culture‐treated flasks in M199 medium supplemented with 20% FBS and 1% penicillin–streptomycin. PCs used were between passages 5 and 10. All cells were incubated at 37°C with 5% CO_2_.

Prior to cell seeding on the electrospun substrates, the fibrillar mats were washed thoroughly with M199 to remove any remaining unreacted polymer, peptide, or photoinitiator. The scaffolds were then sterilized using UV light for 30 min. Confluent flasks of EC and PC were detached from the cell culture substrate using 0.25% trypsin–EDTA (Thermo Fisher Scientific). Cells were seeded by adding cell suspensions dropwise to the scaffolds to form a meniscus and were allowed to adhere for 4 h before gently filling the well with complete medium. To grow an EC‐PC bilayer, the PCs were first seeded as described and allowed to attach for 24 h. The scaffolds were then inverted, and ECs were seeded on the opposite surface using the same methods.

### 2.7 Cell decellularization

Decellularization was achieved using previously described methods.^28^ PC were cultured onto treated 25 mm glass coverslips at a density of 10^6^ cells/mL. Cells were cultured for 14 days with complete medium supplemented with 50 μg/mL ascorbic acid. Decellularization was achieved by removing the cells using 0.5% Triton‐X 100 (Sigma Aldrich) and 10 mM ammonium hydroxide (JT Baker) in PBS for 1 min, followed by treatment with 180 U/mL DNase I (Roche, Basel, Switzerland) for 30 min at 37°C, leaving the ECM on the coverslip.

### 2.8 Mechanical testing

The stiffness of fibers was measured using AFM (Nanowizard 4, Bruker). Samples were measured using either an FS‐1500 cantilever (Oxford Instruments; resonance frequency 1500 kHz, spring constant 6 N/m) or an MSCT probe (Bruker; resonance frequency 22 kHz, spring constant 0.07 N/m) in QI mode. Data were analyzed using the JPK image processing software. Young's modulus was calculated from the force curves using the Hertz–Sneddon model. The indentation threshold used for the Hertz–Sneddon model fit was set at 10% of the sample height. The Poisson ratio was set at 0.5. For the analysis, the calibration of the cantilevers was determined using thermal noise,^29^ and the resonance frequency spectrum was fit with a Lorentz curve.

### 2.9 Fluorescent staining

EC and PC cultured on the fibrillar scaffolds were rinsed with PBS and fixed in 4% paraformaldehyde. The cells were then blocked using 2% bovine serum albumin (BSA; Sigma‐Aldrich) and permeabilized using 0.25% Triton X‐100. Samples were incubated with primary antibodies overnight at 4°C and washed thrice with 2% BSA for 5 min each. Samples were then incubated with secondary antibodies for 1 h at room temperature. The cells were washed with PBS and mounted with Vectashield Antifade Mounting Medium containing DAPI (Vector Labs, Newark, CA, USA). EC were stained for vascular endothelial‐cadherin (VE‐Cadherin; Thermo Fisher Scientific), platelet EC adhesion molecule‐1 (PECAM‐1; Thermo Fisher Scientific), and intercellular adhesion molecule‐1 (ICAM‐1; Thermo Fisher Scientific). PC were stained for neuroglial antigen 2 (NG2; Sigma Aldrich), α‐smooth muscle actin (αSMA; Thermo Fisher Scientific), and platelet‐derived growth factor‐β (PDGFRβ; Santa Cruz). All vascular cells were stained for F‐Actin using Alexa Fluor™ 488 Phalloidin (Thermo Fisher Scientific), collagen IV (Sigma Aldrich), and laminin (Sigma Aldrich). All primary antibodies were labeled with Alexa Fluor™ 488, Alexa Fluor™ 555, and Alexa Fluor™ 647 via appropriate secondary antibodies (Thermo Fisher Scientific).

### 2.10 Confocal microscopy and real‐time imaging

Hydrated electrospun scaffolds were characterized using confocal microscopy. The scaffolds were rendered fluorescent by incorporating 2.5 μl/mL of methacrylated rhodamine into the electrospinning solutions. Fluorescent images of the hydrogels at different PEGDA concentrations, fiber densities, and fiber alignments were acquired using a Zeiss LSM 880 with an Airyscan FAST confocal microscope (Carl Zeiss Microscopy, White Plains, NY, USA). Image analysis and post‐processing were completed using the ZEN (Zeiss) software and ImageJ (NIH, Bethesda, Maryland, USA).

To create a suitable platform for the real‐time observation of neutrophil behavior, the PEG fibrillar meshes were sandwiched between two silicone isolators (Grace Bio Labs, Bend, OR, USA) to eliminate any mechanical influence from the rigid surface of the tissue culture plate on the vascular cells. The sandwiched fibrillar matrix was placed in an in‐house built real‐time microscopy chamber. The chamber setup consisted of a metallic holder, followed by an O‐ring, a glass coverslip, a silicone isolator, a PEG fibrillar matrix, another silicone isolator, a second glass coverslip, another O‐ring, and finally, the bottom metallic holder. When incorporating vascular cells, EC and PC were cultured for 3 days and left untreated or treated with TNF‐α as described above. The cells were then labeled using CellTracker™ Orange (Thermo Fisher Scientific) for 30 min prior to imaging. Neutrophils labeled with CellTracker™ Green were seeded directly onto the scaffolds or vascular cell layers at a concentration of 10^6^ cells/mL. All samples were imaged using a Leica DMi8 (Leica Microsystems Inc., Buffalo Grove, IL, USA). Subsequent image analysis and post‐processing were performed using ImageJ (NIH) and the IMARIS software developed by Oxford Instruments (Abingdon, United Kingdom). Only neutrophils that moved at least 6 μm (∼1/2 cell length) from the origin were included for migration analysis.

### 2.11 SHG imaging and scanning electron microscopy

For SHG imaging, images were acquired on a Zeiss LSM 710 NLO multiphoton microscope. Image analysis was completed using ImageJ and IMARIS. Prior to scanning electron microscopy (SEM), samples were mounted and coated with 8 nm of iridium. SEM images were acquired using a Hitachi SU‐70 scanning electron microscope (Chiyoda City, Tokyo, Japan) with a 5 kV beam.

### 2.12 Statistical analysis

All statistical analysis was completed using Prism 10 (GraphPad Prism, San Diego, CA, USA), except the analysis of AFM measurements, which was carried out using IgorPro (Wavemetrics Inc., Portland, OR, USA), and the correlation analysis, which was performed using the Stats module in Python. One‐way ANOVA with post‐hoc Tukey HSD test was used to assess statistical significance, and significance was set as *p* < 0.05. Outliers beyond two standard deviations from the mean were excluded from the analysis.

### 2.13 Ethics statement

The research described herein was conducted following ethical guidelines and regulations approved by the Human Investigation Committee (HIC) of the Institutional Review Board (IRB) at Yale University, in accordance with the Human Research Protection Program. All protocols for these studies were carried out following the Helsinki Declaration standards. Written informed consent was obtained from all human donors, as outlined in the Yale University HIC IRB protocol number 0902004786.

## 3 RESULTS

### 3.1 Fabrication and characterization of mechanically and architecturally tunable mesh scaffolds

The present study used a complementary approach that integrated principles of polymer chemistry and electrospinning to engineer an in vitro model of the mvBM with customizable biophysical and biochemical properties (Figure 1). PEG, selected for its recognized bioinert characteristics and facile covalent modification, served as the base material for scaffold fabrication. The electrospinning solution comprised PEGs of two distinct molecular weights, 35 and 400 kDa. The inclusion of 400 kDa PEG‐enhanced chain–chain entanglement within the polymer solution, facilitating the formation of uniform fibers. We modified the lower molecular weight PEG to incorporate diacrylate end groups (PEGDA) to serve as an intrafiber stabilizer via photocrosslinking.

Vascular BMs in different tissues exhibit varying degrees of stiffness,^30^, ^31^, ^32^ and the experimental methods employed to collect stiffness measurements can introduce variability into reported values.^33^, ^34^ In addition, the precise characterization of the specialized microvascular ECM becomes even more complex due to the challenges associated with isolating this matrix.^5^, ^28^, ^35^, ^36^ Therefore, here, we used human microvascular PC to create a de novo microvascular BM for direct testing. To generate a human mvBM in vitro, human microvascular PCs were cultured for 14 days with ascorbic acid supplementation. After 14 days, the cultures underwent decellularization, as demonstrated by Brown et al., to preserve cell‐deposited matrix proteins^28^ (Figure 1c). The decellularized matrix remained structurally intact and hydrated, and AFM measurements revealed the Young's modulus of the complex microvascular cell‐deposited ECM to be 0.6 ± 0.3 MPa (mean ± SD). This value served as the lower boundary for the Young's moduli of the engineered scaffolds.

To determine the optimal UV exposure time for fabricating fibrillar networks with stiffness resembling the healthy human mvBM, we treated the electrospun PEG meshes with UV light for 5, 10, or 15 min. Subsequently, the stiffness of the fiber surface was measured using AFM (Figure 1c). A crosslinking duration of 5 min yielded fibers with Young's moduli of 7.0 ± 3 MPa (mean ± SD). Exposure to UV light for 10 min resulted in fibers exhibiting Young's moduli of 10.0 ± 7.5 MPa. Meanwhile, fibers exposed for 15 min were found to have Young's moduli of 13.0 ± 4.0 MPa. The softest matrices (7.0 ± 3 MPa), which most closely emulate the mechanical properties of the healthy mvBM with a synthetic structure capable of preserving its architecture, were selected for all subsequent studies.

PEG meshes of varying fiber diameters were generated by adjusting the PEGDA concentration in the electrospinning solution (Figure 1d). Concentrations ranging from 20% to 30% (w/v) were evaluated, and the morphology and diameter distribution of the resulting scaffolds were quantified (Figure 1e). After incubating the scaffolds in PBS at 37°C for 3 days, z‐stack projections of electrospun fibers at each concentration were captured and quantified using ImageJ. Figure 1e,f depicts the fiber morphology and diameter distribution corresponding to each PEGDA concentration used. All three concentrations yielded uniform, round fibers.

Mean fiber diameters of 423 ± 67 nm (mean ± SD), 586 ± 104 nm, and 777 ± 109 nm were achieved for PEGDA concentrations of 20%, 25%, and 30%, respectively (Figure 1e). To provide context, the diameter of fibers in isolated healthy human placental microvessels was measured from SHG images, revealing an average fiber diameter of 743 ± 376 nm. The measured diameter for 20% PEGDA falls within the lower end of the reported range of the mesh diameter found in the healthy BM and was, therefore, used for all further studies.^5^, ^35^

In addition to fiber diameter, two additional structural parameters of the fibrillar scaffold were modified using electrospinning: fiber orientation and density (Figure 1d). The speed of the rotating collector, which can be set to low, medium, or high settings, determines the orientation of the fiber. At a low speed (348 rpm), isotropic alignment with a uniform angular distribution was observed, while high speed (626 rpm) resulted in anisotropic alignment with a peak at −10°. Medium speed (486 rpm) generated a combination of orientations. Moreover, the density of the electrospun matrices was adjusted by altering the collection time from 5 to 15 min, yielding denser matrices with increased time. These data demonstrate that our system can mimic the thickness of multiple tissue BM and matrix thickening that can occur in disease, given that the thickness of the vascular BM varies from hundreds of nanometers to several microns upon fibrotic remodeling.^36^

### 3.2 Synthetic matrices support vascular cell culture and ECM remodeling events

The vascular walls of precapillary arterioles, capillaries, and postcapillary venules comprise a luminal endothelial layer encompassed by PCs and a BM extending along the vessel wall.^37^ Having validated the tunable architecture and mechanics of PEGDA networks, we next tested whether ECs and PCs could adhere to and grow when cultured on the scaffolds.

After processing PEG solutions into fibrillar form, the subsequent step involved coupling the ubiquitous adhesive ligand Arg‐Gly‐Asp‐Ser (RGDS) to the fiber surface. RGDS conjugation was achieved by reacting monoacrylated PEG and subsequently integrating it into the fibrous matrices, a technique previously utilized in studies involving PEGDA.^38^, ^39^ Although the covalent attachment of other peptides and proteins is possible,^25^ a small and non‐fibrillar adhesive moiety was selected to prevent confounding mechanical contributions of a superimposed network of ECM proteins. Therefore, by directly and uniformly coupling RGDS to the PEGDA fiber matrices, we ensured that the ECM stiffness experienced by the seeded cells was dictated solely by the material itself.^33^

**FIGURE 2:**
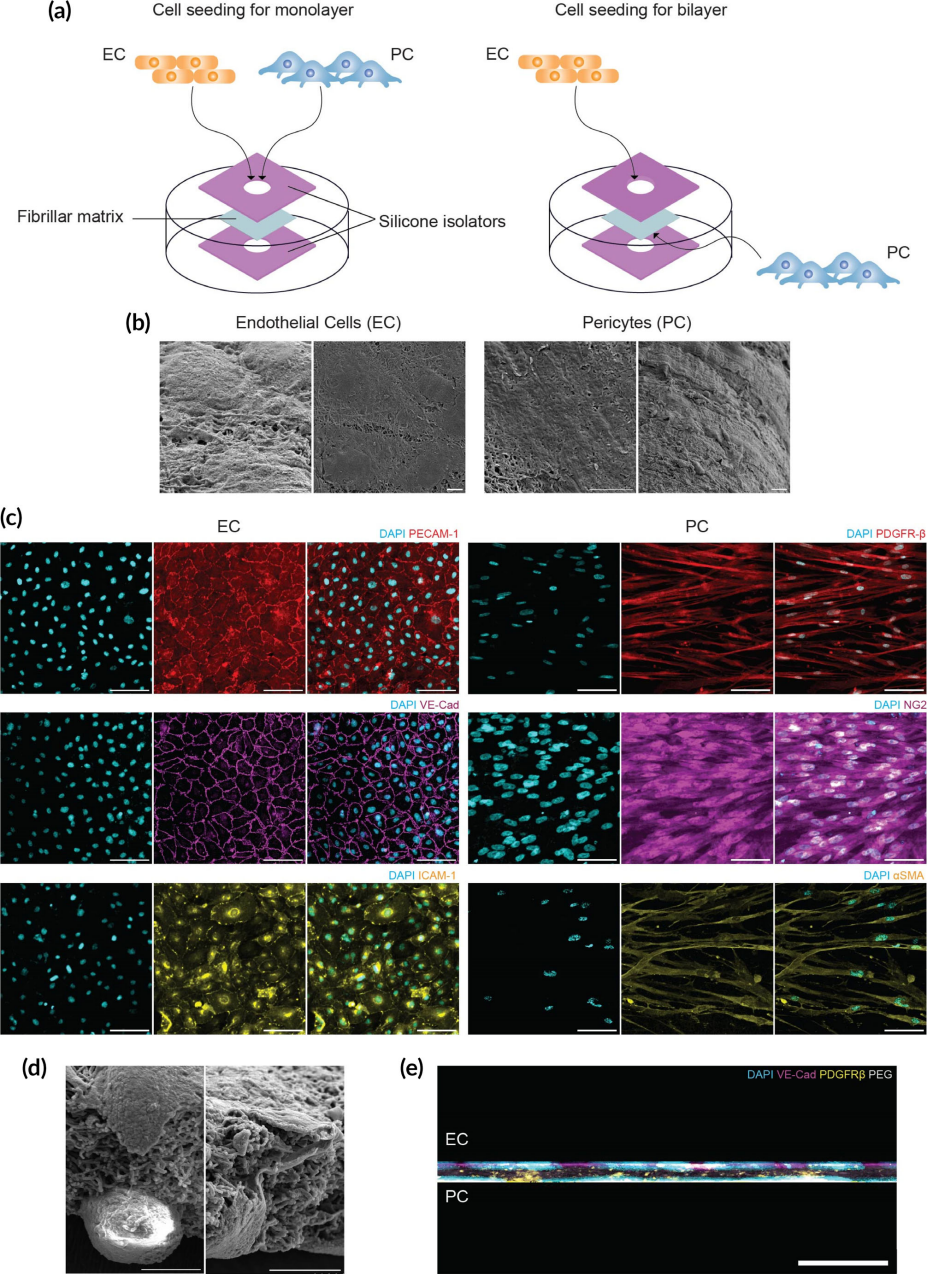
Vascular cell culture on synthetic matrices in mono‐ and bilayer configurations. (a) Diagram of the cell seeding strategy. Soft matrices with RGDS coating conjugation were placed between dual silicone isolators to negate mechanical impact from the rigid tissue culture plate on EC and PC. (b) Scanning electron micrographs of EC and PC seeded on fibrillar matrices. Scale bar: 10 μm. (c) Confocal microscopy images of HUVEC (EC) cultured on soft fibrillar matrices, immunostained for VE‐Cadherin (magenta), PECAM‐1 (red), ICAM‐1 (yellow), and counterstained with DAPI (cyan). Confocal microscopy images of PC cultured on soft matrices, immunolabeled for NG2 (magenta), PDGFRβ (red), αSMA (yellow), and DAPI (cyan). Scale bar: 100 μm. (d) Cross‐sectional SEM image of EC‐PC bilayer seeded on fibrillar matrices. Scale bar: 10 μm. (e) Confocal view of EC and PC seeded in a bilayer arrangement. Cells are marked with DAPI (cyan), VE‐Cadherin (EC, magenta), and PDGFRβ (PC, yellow). The PEG fiber network is labeled with rhodamine‐methacrylate (white). Scale bar: 50 μm.

Soft matrices coated with RGDS were sandwiched between two silicone isolators to eliminate any mechanical influence from the rigid surface of the tissue culture plate on the vascular cells (Figure 2a). Scanning electron micrographs confirmed the adhesion and growth of EC and PC seeded on the soft fibrillar matrices (Figure 2b). We utilized HUVECs to test the formation of an endothelium on the fibrillar scaffolds. We established that HUVECs adhere to the surface of the fibers, creating a monolayer atop the scaffold. Further imaging suggests that fiber directional alignment can influence EC orientation (Figure S1). Immunofluorescent staining confirmed the expression of essential proteins involved in maintaining the endothelial lumen and a baseline level of adhesion molecule expression, namely VE‐Cadherin, PECAM‐1, and ICAM‐1 (Figure 2c).

Following a culture period of 3–4 days on PEGDA matrices, the HUVECs exhibited VE‐Cadherin expression, indicating the formation of a selectively permeable endothelial barrier.^40^ The presence of PECAM‐1 at intercellular junctions further substantiated the integrity of the endothelial monolayer.^41^ Lastly, ICAM‐1 expression on the surface of cultured HUVECs validated that ECs cultured on fibrillar matrices express proteins capable of responding adequately to inflammatory stimulation.^41^, ^42^

As a source of mural cells, we used primary PCs isolated from healthy human placentas. Unlike ECs, mural PCs do not form a traditional monolayer but maintain a non‐overlapping spatial domain along the microvasculature.^42^ PCs depend heavily on PDGFβ signaling for their retention along vessels.^42^, ^43^, ^44^ As such, we validated PDGF receptor‐β (PDGFRβ) expression on the surface of PCs cultured on fibrillar matrices (Figure 2c). After 3–4 days in culture, PCs maintain their expression of PDGFRβ. Similarly, immunofluorescence staining confirmed the expression of NG2 and αSMA, two proteins commonly expressed by microvascular PCs. These results demonstrate that EC and PC culture is supported by fibrillar PEGDA networks that express critical proteins involved in vascular homeostasis.

Furthermore, SEM demonstrated our ability to culture EC and PC as a bilayer, as depicted in the cross‐section micrographs shown in Figure 2d. Confocal microscopy further confirmed the creation of the bilayer system and, thus, the formation of an in vitro composite microvascular wall structure (Figure 2e).

**FIGURE 3:**
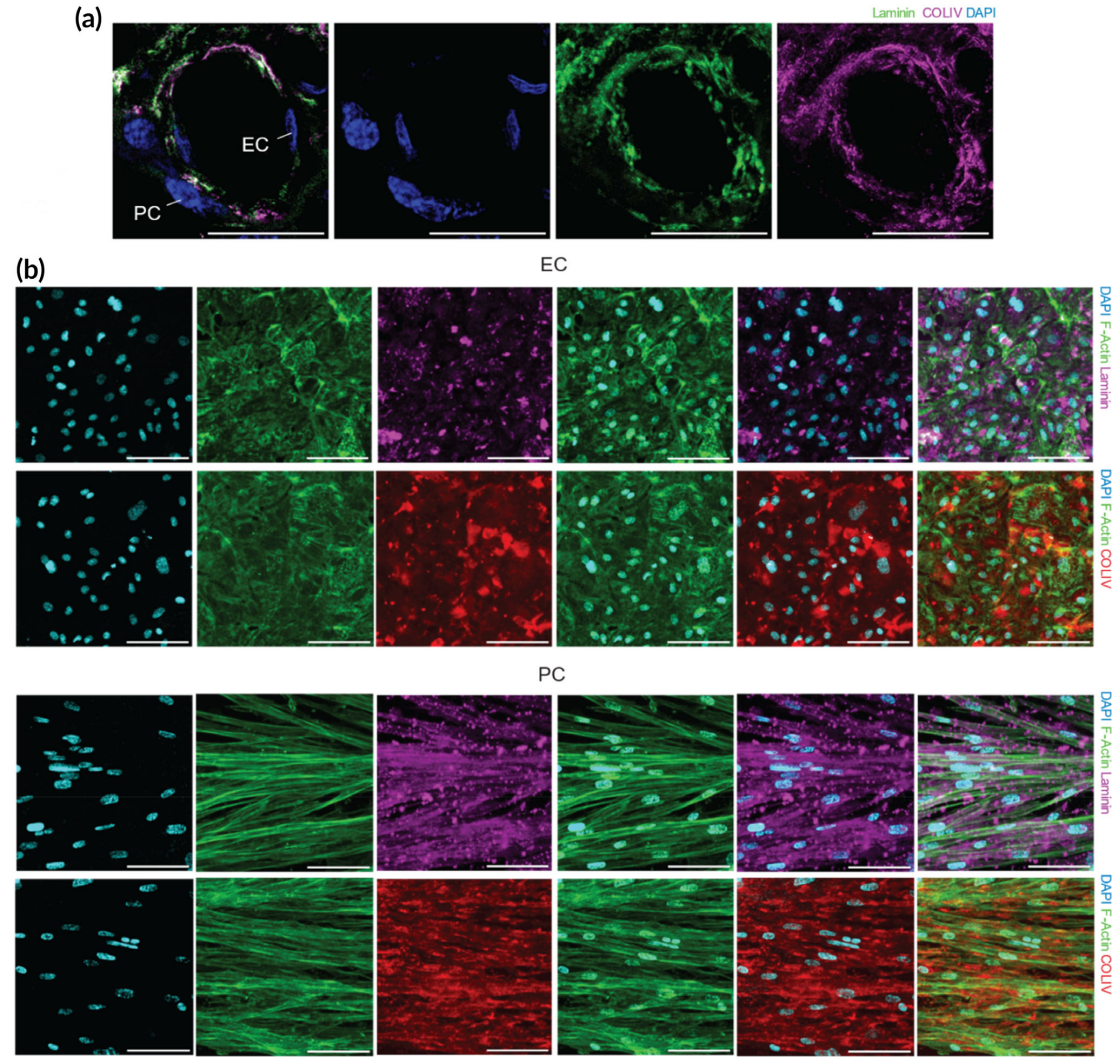
ECM remodeling induced by vascular cells on PEG fibrillar matrices. (a) A representative slice of a healthy human placental microvessel, immunostained for laminin (green), collagen IV (COLIV, magenta), and DAPI (blue). Scale bar: 20 μm. (b) Confocal images of cultured EC and PC, immunofluorescently stained for ECM proteins: Laminin (magenta) and COLIV (red). The co‐localization of the cell cytoskeleton (F‐Actin, green) with these markers indicates ECM deposition around and beneath the cells. Scale bar: 100 μm.

Having successfully reconstructed a microvascular wall with tunable architecture and mechanics, inclusive of ECs and PCs, and a mvBM mimetic matrix, we sought to determine whether this complex structure could facilitate ECM remodeling by the incorporated cells. The major components that constitute the structure of the mvBM are laminin and collagen IV (Figure 3a).^45^ To assess the ability of cultured ECs and PCs to deposit mvBM proteins, we utilized immunofluorescent staining of these matrix proteins. Notably, only extracellular proteins were stained to observe matrix deposition specifically. Confocal imaging confirmed laminin and collagen IV deposition by both cell types (Figure 3b). Additional cross‐sectional microsopy revealed that the matrix proteins deposited by ECs and PCs cultured on our synthetic matrix were distributed on and beneath the cellular bodies, as indicated by the overlap of laminin and collagen IV deposition with the cytoskeleton of the cells (Figure S2). These results suggest that our synthetic matrix supports cell adhesion and growth of vascular cells and promotes ECM remodeling by facilitating the deposition of key mvBM proteins. This further highlights the potential of our system as a physiologically relevant model for studying microvascular wall remodeling dynamics in vitro.

### 3.3 mvBM mimetic allows for real‐time capture of leukocyte migration events

**FIGURE 4:**
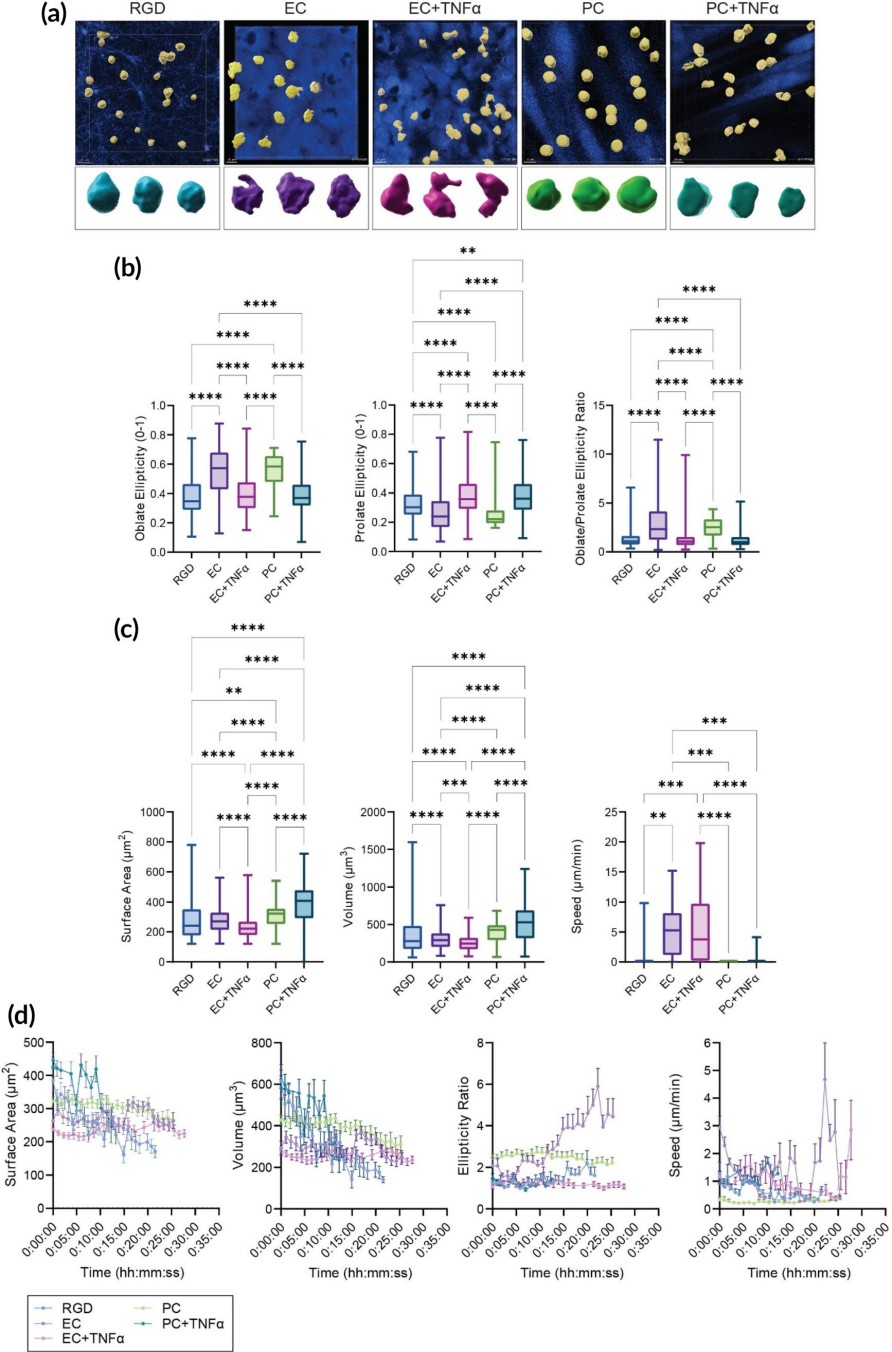
Analysis of neutrophil migration in the microvascular wall. (a) Confocal microscopy images of neutrophils seeded on different experimental substrates. These images highlight the interaction of neutrophils with the substrates, providing visual evidence of their morphological adaptations and migration patterns under different experimental conditions. (b) Box and whiskers plots delineating the oblate ellipticity, prolate ellipticity, and the ratio of oblate‐to‐prolate ellipticity of neutrophils. The data is segmented based on different seeding conditions: Neutrophils on a fiber mesh alone and those in contact with EC or PC, with and without TNFα stimulation. Statistical analysis was performed using one‐way ANOVA. Significance levels are indicated as follows: **p ≤ 0.01; **** *p* ≤ 0.0001. (c) Box and whiskers plot of surface area (SA), volume, and speed of neutrophils under the same experimental setups as in Panel (a). (d) Plots of the average surface area, volume, ellipticity ratio, and speed of neutrophils over time, again under the varied conditions of being seeded on a fiber mesh alone and in the presence of EC or PC, with and without TNFα stimulation.

Pathological neutrophil recruitment, implicated in the progression of various diseases, is influenced by dysregulated changes to the mvBM.^16^ To elucidate the impact of microenvironmental parameters on neutrophil recruitment, we have integrated our modular mimetic of the mvBM with a real‐time microscopy chamber (Figure S3). This configuration allows for dynamic profiling and real‐time visualization of neutrophil recruitment, providing insights into how changes in the vascular microenvironment can affect neutrophil behavior.^46^ We cultured vascular cells, labeled with CellTracker™ Orange, on the scaffolds and exposed them to TNF‐α treatment (20 ng/mL) or left them untreated for 4 h. We then seeded neutrophils, isolated from healthy human donors, labeled with CellTracker™ Green, onto EC or PC layers cultured on fibrillar matrices or directly onto the RGD‐coated scaffolds. Using confocal microscopy, we acquired real‐time 3D images under all experimental conditions. These images were subsequently analyzed using IMARIS software to quantify the ellipticity, surface area (SA), volume, and speed of neutrophils, providing insights into their morphology and motility in response to our vascular mimetic (Figure 4a).

In this study, ellipticity was utilized as a quantitative metric to assess deviations in cellular morphology from a spherical baseline, facilitating the evaluation of cellular responses under various experimental conditions. This metric was dimensionally represented on a scale from 0 to 1, where a value of 0 corresponded to an ideal spherical form, while a value approaching 1 indicated a significant deviation toward either oblateness or prolateness. Oblateness represents a flattening primarily along the horizontal x,y‐plane, whereas prolateness denotes an elongation predominantly along the vertical z‐plane.

For neutrophils seeded on fibrillar matrices alone, oblate ellipticity was 0.3775 ± 0.1298 (mean ± SD) (Figure 4b). Neutrophils seeded on unactivated EC and PC exhibited oblate ellipticity values of 0.5537 ± 0.1613 and 0.5544 ± 0.1125, respectively. Upon TNF‐α activation, oblate ellipticity values decreased to 0.3935 ± 0.1132 for neutrophils on EC and 0.3906 ± 0.1181 for neutrophils on PC. The reduction in oblate ellipticity for neutrophils seeded on activated vascular layers, indicative of a lesser degree of flattening post‐activation, was statistically significant (*p* < 0.0001 for neutrophils on both EC and PC treated vs. untreated cells).

Prolate ellipticity for neutrophils seeded on fibrillar matrices alone was 0.3315 ± 0.1147. For neutrophils interacting with unactivated EC and PC, prolate ellipticity decreased to 0.2745 ± 0.1498 and 0.2597 ± 0.1056, respectively. After TNF‐α activation, prolate ellipticity increased to 0.3799 ± 0.1277 for EC and 0.3763 ± 0.1380 for PC. This shift toward greater elongation following activation was statistically significant (*p* < 0.0001 for both).

The oblate‐to‐prolate ellipticity ratio, indicative of overall cell shape with a ratio of 1 indicating overall sphericity, was 1.306 ± 0.7944 (mean ± SD) for neutrophils on the scaffold alone. Neutrophils seeded on TNF‐α‐treated vascular cells displayed similar ellipticity ratios, with values of 1.226 ± 0.7716 on EC and 1.242 ± 0.782 on PC. Conversely, a significant increase in the oblate‐to‐prolate ellipticity ratio was observed for neutrophils seeded on untreated vascular cells, with values of 2.985 ± 2.283 for EC and 2.476 ± 0.9889 for PC (*p* < 0.0001), indicating a more two‐dimensional, or lateral, migratory pattern.

The SA of neutrophils seeded on different substrates varied, with values of 281 ± 133.4 μm^2^ for cells directly onto fibers (Figure 4c). Neutrophils on quiescent EC and PC displayed SAs of 274.1 ± 82.33 μm^2^ and 305.7 ± 76.72 μm^2^, respectively. TNF‐α activation resulted in neutrophil SAs of 234.5 ± 76.77 μm^2^ for cells seeded on EC and 391.9 ± 136.2 μm^2^ for cells seeded on PC. Notably, TNF‐α‐activated PC showed a statistically significant increase in neutrophil SA compared to the untreated control, while unactivated and TNF‐α‐activated EC conditions exhibited the opposite trend. Neutrophil volumes mirrored neutrophil SA trends, with values of 378.1 ± 284.2 μm^3^ for neutrophils on the scaffold alone, 298 ± 128.2 μm^3^ for untreated EC, and 389.4 ± 135 μm^3^ for neutrophils on untreated PC. TNF‐α activation resulted in volumes of 255.9 ± 107.5 μm^3^ for neutrophils on EC and 517.9 ± 253.1 μm^3^ for neutrophils on PC.

Neutrophil migration speeds varied across substrates (Figure 4c), with the highest observed for neutrophils on TNF‐α‐treated EC at 6.00 ± 1.38 μm/min, followed by neutrophils on untreated EC at 4.98 ± 0.92 μm/min, and neutrophils on PEG fibers at 0.82 ± 0.58 μm/min. Neutrophils on untreated PC migrated at 0.27 ± 0.01 μm/min, with a minimal increase on TNF‐α‐treated PC at 1.076 ± 0.06 μm/min. The lower speeds observed in neutrophils on cultured PC align with reports of neutrophils necessitating EC transmigration for efficient neutrophil trans‐PC traversal.^47^

Additionally, we found a statistically significant (*p* < 0.0001) negative correlation between cell SA and volume and track straightness (also termed persistence), indicating that rounder, more compact neutrophils migrating on TNFα‐activated EC are migrating more persistently (Figures S4 and S5). Overall, the changes in morphology and motility we observed align with the rounder and more compact morphology and motility trends of in vivo migrating neutrophils.^37^

To comprehensively understand the dynamic behavior of neutrophils during each step of vascular transmigration, we also conducted a real‐time analysis of each of the aforementioned parameters (Figure 4d). This allowed us to observe and quantify the temporal variations in these parameters, providing valuable insights into the changing behavior of neutrophils over time. Higher fluctuations in SA and volume were observed for neutrophils seeded directly onto the fibrillar matrices or neutrophils seeded on PC treated with TNFα. Interestingly, fluctuations in the oblate‐to‐prolate ratio of neutrophils were generally muted under most conditions. However, we noted a rapid and significant increase in oblateness over time for neutrophils seeded on inactivated EC.

Regarding speed, we observed higher fluctuations over time in neutrophils interacting with EC than in those seeded on the PC or the scaffolds. This could potentially reflect the dynamic nature of neutrophil–EC interactions, which involve rapid changes in adhesion, deformation, and propulsion forces.^48^, ^49^ Notably, frames from timelapse capture demonstrated that TNFα‐treated PC were highly contractile (Figure S6). Adherent and motile neutrophils appeared to collect in the gaps created by PC contraction, suggesting a dynamic interaction between neutrophils and PC under inflammatory conditions. Our observations provide a glimpse into the swift changes in neutrophil shape and motility during vascular transmigration, that coincide with EC and PC engagement. These findings can complement transcriptomic and proteomic profiling of neutrophils in diverse, dynamic biological settings.^46^

## 4 DISCUSSION

The mvBM has emerged as an integral contributor to maintaining vascular integrity and orchestrating cellular functions, making it a crucial area of study in the field of vascular biology. Despite widespread acknowledgment of its importance, the intricate mechanisms through which the mvBM influences health and disease remain to be fully described, particularly in pathologies characterized by pronounced vascular dysregulation and abnormal neutrophil recruitment. While existing literature offers insights into the biochemical composition and biological activities of the mvBM, it is only recently that the importance of its physical properties, like mechanical stiffness and topography, have gained recognition.^11^ This growing area of investigation highlights a significant knowledge gap: our understanding of the mvBM's integral role in vascular homeostasis and disease pathogenesis is still in its infancy, exacerbated by the inherent challenges in isolating this specialized type of ECM. In vitro models of the mvBM thus present a unique avenue for dissecting and understanding the individual biochemical and biomechanical factors and their respective contributions to overall function, with significant implications for developing targeted therapies. However, existing models often focus on individual elements, thereby overlooking critical components such as the role of the microenvironment in modulating vascular cell behavior and the integration of key players like mural PCs. This situation underscores a pressing need to develop innovative, controlled, and comprehensive microenvironments that not only emulate physiologically relevant conditions but also offer the capability for real‐time monitoring of cellular dynamics.^11^

This study introduces an engineered in vitro mvBM mimetic characterized by versatile biophysical and biochemical parameters that can replicate healthy and disease states. Advanced imaging techniques, including SHG imaging and AFM, were employed to characterize the human microvascular BM's morphology and mechanical properties, guiding the design constraints of our in vitro system. In the development of a novel in vitro model of the mvBM, we used PEG to create a versatile system that allows for the replication of various aspects of the mvBM, including mechanical stiffness, fiber diameter, orientation, and density, providing a valuable tool for studying physiological and pathological states. This engineered model supports the culture of human EC and PC along with their own deposited ECM proteins, facilitating the formation of a composite microvascular wall within a controlled environment, thus enabling detailed studies of microvascular pathology and potential therapeutic interventions. Our approach integrates real‐time microscopy, allowing for the dynamic profiling of neutrophil responses to the scaffold itself, as well as the cultured vascular EC and PC, in a temporally relevant manner.

Functionalization of RGDS on the surface of the PEG‐based fibrous meshes facilitated the culture of ECs and PCs, as evidenced by the robust expression of essential proteins associated with endothelial function (e.g., VE‐Cad, PECAM‐1, and ICAM‐1) and PC function (e.g., PDGFRβ, NG2, and αSMA). Immunofluorescent staining corroborated the deposition of laminin and collagen IV, essential constituents of the in vivo mvBM, by both ECs and PCs on the synthetic matrix. These findings affirm the efficacy of our engineered system in supporting cellular adhesion, growth, and ECM remodeling, underscoring its potential as a physiologically relevant in vitro model for investigating microvascular wall dynamics and for screening potential therapeutics. The ability of these scaffolds to support vascular cell matrix deposition provides the additional opportunity to evaluate the role of microvascular matrix remodeling in vascular dysfunction, capillary rarefaction, and other pathological effects of the matrix on largely understudied peripheral vasculature.

In addition, we demonstrated our ability to integrate our novel mvBM model with 4D microscopy, thereby broadening the applicability of our system for the investigation of microvascular dynamics and the real‐time interactions between neutrophils and the engineered environment. Our results unveil distinct and dynamic neutrophil behaviors in response to their interactions with various components of the vascular wall—EC, PC, and BM—both in the presence and absence of inflammatory stimulation. Specifically, when interacting with TNFα‐treated EC or PC, neutrophils exhibited an increased propensity to transmigrate, as evidenced by their augmented tendency to elongate and probe in the z‐direction. Conversely, when exposed to untreated EC or PC, the neutrophils demonstrated greater cell spreading and 2D lateral probing indicative of random sampling. As expected, neutrophils interacting with TNFα‐stimulated EC had the fastest migration speed. This observation is consistent with previous reports of rapid neutrophil migration along the EC layer,^50^ thereby locating an optimal site for transendothelial migration, subsequently being primed by the endothelium to more efficiently interact with the adhesion molecules presented by the PC layer.^47^

Dynamic sampling of neutrophil behaviors in various microenvironmental contexts facilitated the collection of results, leading to the identification of correlations between multiple parameters. Our results revealed an association between a rounder and more compact phenotype and heightened migratory persistence, aligning with extant in vivo reports of migratory neutrophils in zebrafish.^37^ This observed phenomenon is also in line with reports of neutrophils in sepsis, where neutrophils that tend to be larger and thus more rigid have diminished migratory and chemotactic capabilities.^51^ This multiparametric characterization of neutrophil behavior has been emphasized as a valuable complement and, in some cases, superior to proteomic and transcriptomic profiling, particularly in highly dynamic scenarios where transient cell states dictate biological function, as identifying these behavioral phenotypes can improve our understanding of neutrophil physiology.^46^, ^51^ Subsequently, this knowledge has the potential to steer the development of novel therapeutic strategies.

Moreover, the temporal analysis of neutrophil morphology and behavior in this study provides valuable insights into their interactions with various substrates, capturing rapid transitions and transient behaviors that static measurements would overlook. For example, while neutrophils on RGD‐coated fibers displayed oblate‐to‐prolate ratios similar to those on TNF‐α‐treated ECs and PCs, the integration of morphological data with motility and temporal dynamics revealed critical differences in their behavior. Neutrophils on TNF‐α‐activated ECs and PCs exhibited significantly higher migration speeds than those on scaffolds alone. However, the slightly higher speed observed on TNF‐α‐treated PCs compared to the scaffold was not statistically significant, consistent with previous findings that neutrophils require EC priming to efficiently transmigrate across the PC layer.^47^ Additionally, temporal morphological analysis showed that neutrophils on scaffolds experienced lower fluctuations in shape compared to those interacting with ECs or PCs, reflecting a more static interaction in the absence of inflammatory activation cues.

The investigations outlined in this study represent only a fraction of the system's capacity for understanding the intricate interactions between each component of the microvascular wall and its role in acute and chronic inflammation. While the application of TNFα served as evidence for an inflammatory response within our model, there is a need for further exploration involving other proinflammatory cytokines. For instance, previous studies reported that different adhesive proteins can mediate neutrophil transmigration in vivo in a stimulus‐dependent manner.^52^, ^53^ This is particularly important as our observed neutrophil velocities were slower than those reported in vivo,^54^ a phenomenon likely attributable to the complex cascade of pro‐migratory stimuli in the in vivo setting and the lack of vascular flow. Despite this difference, our model is an improvement over previous systems; however, its fidelity can be further enhanced by integrating shear flow. Accordingly, future efforts will be directed toward incorporating laminar flow into the model.

Despite these differences, our model represents a significant improvement over previous systems. However, its physiological relevance can be further enhanced by incorporating shear flow, a key determinant of vascular cell behavior. Shear stress is critical for EC alignment, cytoskeletal organization, and the regulation of cellular functions, such as adhesion molecule expression, which are essential for neutrophil recruitment and transmigration.^55^, ^56^, ^57^, ^58^ PCs are similarly responsive to biomechanical cues, and the interplay between flow and matrix topography could synergistically influence their alignment and functionality. Integrating laminar flow into future iterations of the model would enable the evaluation of how neutrophil interactions with the microvascular wall are modulated by the combined effects of fiber orientation, EC and PC alignment, and flow‐induced forces. This advancement would provide crucial insights into the complex interplay of mechanical and biochemical signals in both physiological and pathological states, further enhancing the system's utility as a comprehensive tool for studying vascular dynamics.

Lastly, it is vital to recognize that our present model, employing tissue‐matched human EC and PC, provides an opportunity to investigate organ‐specific mechanisms of neutrophil recruitment, which have been demonstrated to exhibit variation across different organs.^59^

## 5 CONCLUSIONS AND PERSPECTIVES

In summary, our study introduces an engineered in vitro model replicating the human mvBM to investigate real‐time microvascular dynamics and neutrophil interactions. This innovative model provides precise control over the biochemical and biophysical properties of the mvBM, enabling the decoupling and examination of individual factors in vascular function. The versatility of our model allows for the replication of various disease states, providing a robust platform to identify novel therapeutic targets in vascular‐related diseases and serving as a tool in preclinical studies of new treatments. The coupling of this system with advanced transcriptomic and proteomic methodologies holds the potential to facilitate the identification of key biological functions and behaviors in specific vascular microenvironments. These capabilities are crucial for understanding the mechanisms underlying vascular‐related diseases like diabetes, fibrosis, and inflammatory disorders.

Furthermore, this system can be used to explore organ‐specific vascular responses, advancing our understanding of systemic diseases and enhancing the development of targeted therapies. Overall, this research represents a major advancement in vascular biology research, poised to translate basic discoveries into potential clinical applications.

## AUTHOR CONTRIBUTIONS

**Laura C. Morales:** Investigation; conceptualization; funding acquisition; writing – original draft; methodology; formal analysis; data curation; writing – review and editing. **Catherine D. Kim:** Investigation; writing – review and editing. **Yangang Pan:** Validation; conceptualization; visualization; formal analysis; writing – review and editing; methodology. **Simon Scheuring:** Validation; conceptualization; visualization; formal analysis; supervision; resources; writing – review and editing; methodology. **Anjelica L. Gonzalez:** Conceptualization; investigation; funding acquisition; supervision; resources; formal analysis; project administration; writing – review and editing; methodology.

## CONFLICT OF INTEREST STATEMENT

The authors declare no conflicts of interest.

## PEER REVIEW

The peer review history for this article is available at https://www.webofscience.com/api/gateway/wos/peer-review/10.1002/btm2.70008.

## Supporting information


**Figure S1.** EC alignment to fiber orientation in PEG matrices. Confocal images show EC alignment along anisotropic fiber orientations (top panel) and lack of alignment in isotropic fiber orientations (bottom panel). ECs are stained for F‐actin (yellow) and nuclei (DAPI, magenta), while PEG fibers are fluorescently labeled with rhodamine‐MA (cyan). Scale bar: 20 μm.
**Figure S2.** Top and cross‐sectional 3D views of immunofluorescent staining of ECM deposited by ECs and PCs cultured on a PEG fibrillar matrix. Confocal images show ECM proteins COLIV (magenta) and laminin (red), alongside the cell cytoskeleton (F‐actin, green) and PEG fibers (gray). The co‐localization of ECM proteins with the cell cytoskeleton highlights ECM deposition around and beneath the cells. Scale bar: 50 μm.
**Figure S3.** Schematic Representation of Multi‐Layered In‐House Built Customized Microscopy Chamber for Real‐Time Neutrophil Migration Studies. (A) An exploded schematic view of the microscopy chamber assembly. The diagram delineates the sequential arrangement of the chamber's components, starting from the top with a metallic holder, followed by an O‐ring, glass coverslip, silicone isolator, a PEG fibrillar matrix, another silicone isolator, a second glass coverslip, another O‐ring, and finally, the bottom metallic holder. (B) This panel is divided into two views of the assembled microscopy chamber: a top‐down view and a cross‐sectional view. The top‐down view offers a focused look at the concentrically layered structure and the central well where neutrophils are placed for migration studies. The cross‐sectional view reveals the spatial arrangement of the layers within the chamber.
**Figure S4.** Correlation Analysis of Neutrophil Morphometrics and Kinetics from Compiled Neutrophils 4D Microscopy Data. (A) Correlation matrix that illustrates both positive and negative correlation coefficients across a range of measured variables obtained with IMARIS. These variables include the surface area (Area), volume, oblate and prolate ellipticities, track straightness, surface area‐to‐volume (SAV) ratio, and the oblate‐to‐prolate ellipticity ratio. Each cell within the matrix represents the degree of correlation between any two variables, with color intensity and/or numerical values indicating the strength and direction of the correlation. (B) Statistical significance of the correlations between key parameters (p‐values shown). Highlighted are the statistically significant relationships between surface area and volume, as well as between track straightness and the SAV ratio in migrating neutrophils across all experimental conditions.
**Figure S5.** Additional Parameters Obtained from IMARIS Analysis of 4D Neutrophil Migration Data. Box and whiskers plots illustrate three additional parameters of neutrophil migration: track displacement, length, and straightness. These metrics are compared across neutrophils seeded on a standalone fibrillar matrix versus those seeded on vascular cells, with further subgroup analysis based on the presence or absence of TNFα activation. One‐way ANOVA was used to assess the significance of differences observed across conditions. Significance levels are denoted with asterisks representing p‐values as follows: * (*p* ≤ 0.05), ** (*p* ≤ 0.01), *** (*p* ≤ 0.001), **** (*p* ≤ 0.0001).
**Figure S6.** Preliminary observations of PC activated with TNF‐α during interactions with neutrophils. Confocal microscopy time‐lapse imaging captures PC (blue) contractility in real‐time as they interact with neutrophils (yellow). Scale bar: 10 μm.

## Data Availability

The data that support the findings of this study are available from the corresponding author upon reasonable request.
