# Supplementary material for: Engineered microvascular basement membrane mimetic for real‐time neutrophil tracking in the microvascular wall
Source: Bioeng Transl Med. 2025 Mar 12;10(4):e70008. doi: 10.1002/btm2.70008 (PMC12284441; doi:10.1002/btm2.70008)
Supplement: Supplementary file 1 — Figure S1. EC alignment to fiber orientation in PEG matrices. Confocal images show EC alignment along anisotropic fiber orientations (top panel) and lack of alignment in isotropic fiber orientations (bottom panel). ECs are stained for F‐actin (yellow) and nuclei (DAPI, magenta), while PEG fibers are fluorescently labeled with rhodamine‐MA (cyan). Scale bar: 20 μm. Figure S2. Top and cross‐sectional 3D views of immunofluorescent staining of ECM deposited by ECs and PCs cultured on a PEG fibrillar matrix. Confocal images show ECM proteins COLIV (magenta) and laminin (red), alongside the cell cytoskeleton (F‐actin, green) and PEG fibers (gray). The co‐localization of ECM proteins with the cell cytoskeleton highlights ECM deposition around and beneath the cells. Scale bar: 50 μm. Figure S3. Schematic Representation of Multi‐Layered In‐House Built Customized Microscopy Chamber for Real‐Time Neutrophil Migration Studies. (A) An exploded schematic view of the microscopy chamber assembly. The diagram delineates the sequential arrangement of the chamber's components, starting from the top with a metallic holder, followed by an O‐ring, glass coverslip, silicone isolator, a PEG fibrillar matrix, another silicone isolator, a second glass coverslip, another O‐ring, and finally, the bottom metallic holder. (B) This panel is divided into two views of the assembled microscopy chamber: a top‐down view and a cross‐sectional view. The top‐down view offers a focused look at the concentrically layered structure and the central well where neutrophils are placed for migration studies. The cross‐sectional view reveals the spatial arrangement of the layers within the chamber. Figure S4. Correlation Analysis of Neutrophil Morphometrics and Kinetics from Compiled Neutrophils 4D Microscopy Data. (A) Correlation matrix that illustrates both positive and negative correlation coefficients across a range of measured variables obtained with IMARIS. These variables include the surface area (Area), v [file BTM2-10-e70008-s001.docx]

**Supplemental Figures**


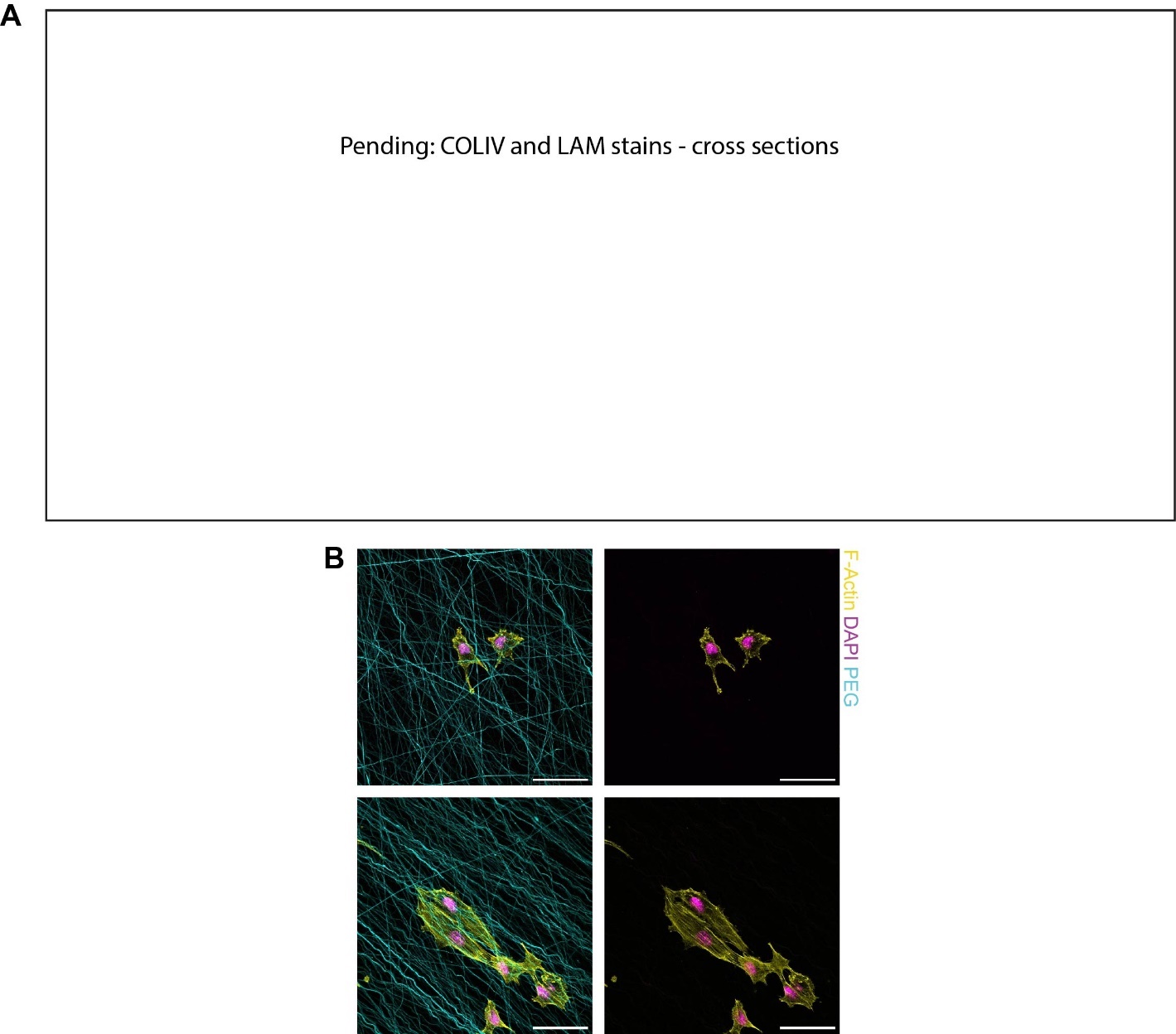


**Supplemental Figure 1. EC alignment to fiber orientation in PEG matrices.** Confocal images show EC alignment along anisotropic fiber orientations (top panel) and lack of alignment in isotropic fiber orientations (bottom panel). ECs are stained for F-actin (yellow) and nuclei (DAPI, magenta), while PEG fibers are fluorescently labeled with rhodamine-MA (cyan). Scale bar: 20 µm.


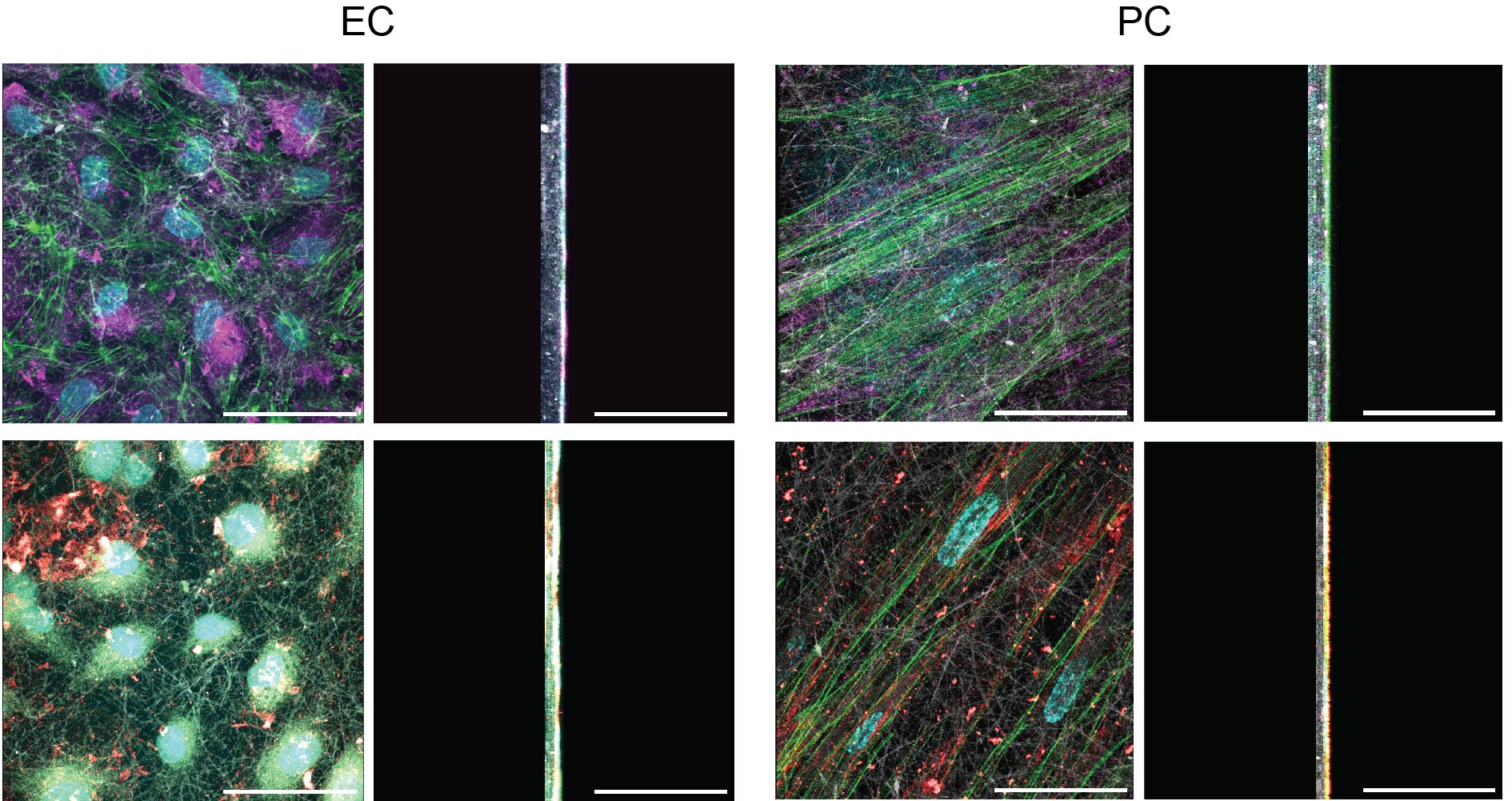


**Supplemental Figure 2. Top and cross-sectional 3D views of immunofluorescent staining of ECM deposited by ECs and PCs cultured on a PEG fibrillar matrix.** Confocal images show ECM proteins COLIV (magenta) and laminin (red), alongside the cell cytoskeleton (F-actin, green) and PEG fibers (gray). The co-localization of ECM proteins with the cell cytoskeleton highlights ECM deposition around and beneath the cells. Scale bar: 50 µm.


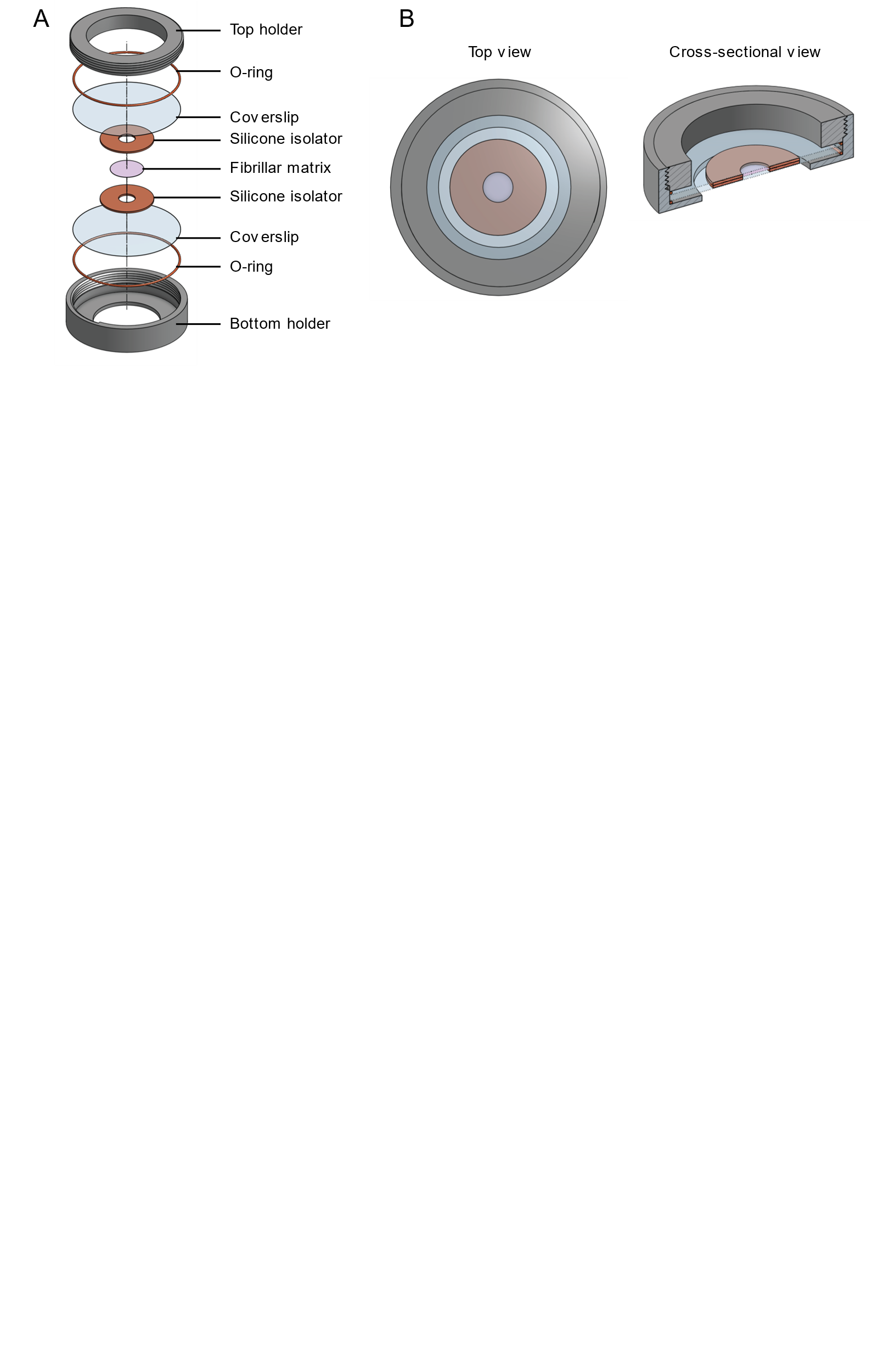


**Supplemental Figure 3. Schematic Representation of Multi-Layered In-House Built Customized Microscopy Chamber for Real-Time Neutrophil Migration Studies.** (A) An exploded schematic view of the microscopy chamber assembly. The diagram delineates the sequential arrangement of the chamber’s components, starting from the top with a metallic holder, followed by an O-ring, glass coverslip, silicone isolator, a PEG fibrillar matrix, another silicone isolator, a second glass coverslip, another O-ring, and finally, the bottom metallic holder. (B) This panel is divided into two views of the assembled microscopy chamber: a top-down view and a cross-sectional view. The top-down view offers a focused look at the concentrically layered structure and the central well where neutrophils are placed for migration studies. The cross-sectional view reveals the spatial arrangement of the layers within the chamber.


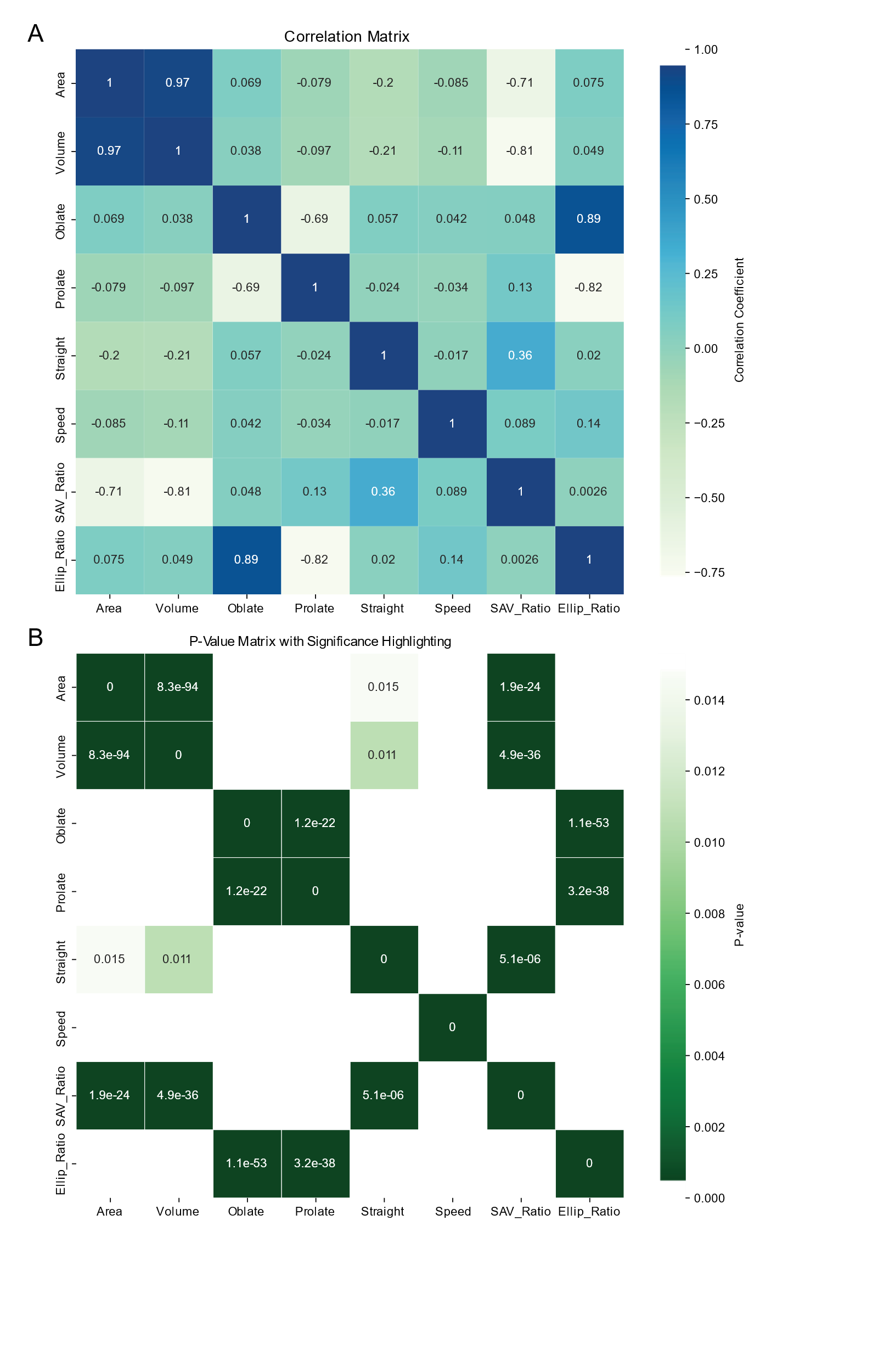


**Supplemental Figure 4. Correlation Analysis of Neutrophil Morphometrics and Kinetics from Compiled Neutrophils 4D Microscopy Data.** (A) Correlation matrix that illustrates both positive and negative correlation coefficients across a range of measured variables obtained with IMARIS. These variables include the surface area (Area), volume, oblate and prolate ellipticities, track straightness, surface area-to-volume (SAV) ratio, and the oblate-to-prolate ellipticity ratio. Each cell within the matrix represents the degree of correlation between any two variables, with color intensity and/or numerical values indicating the strength and direction of the correlation. (B) Statistical significance of the correlations between key parameters (p-values shown). Highlighted are the statistically significant relationships between surface area and volume, as well as between track straightness and the SAV ratio in migrating neutrophils across all experimental conditions.


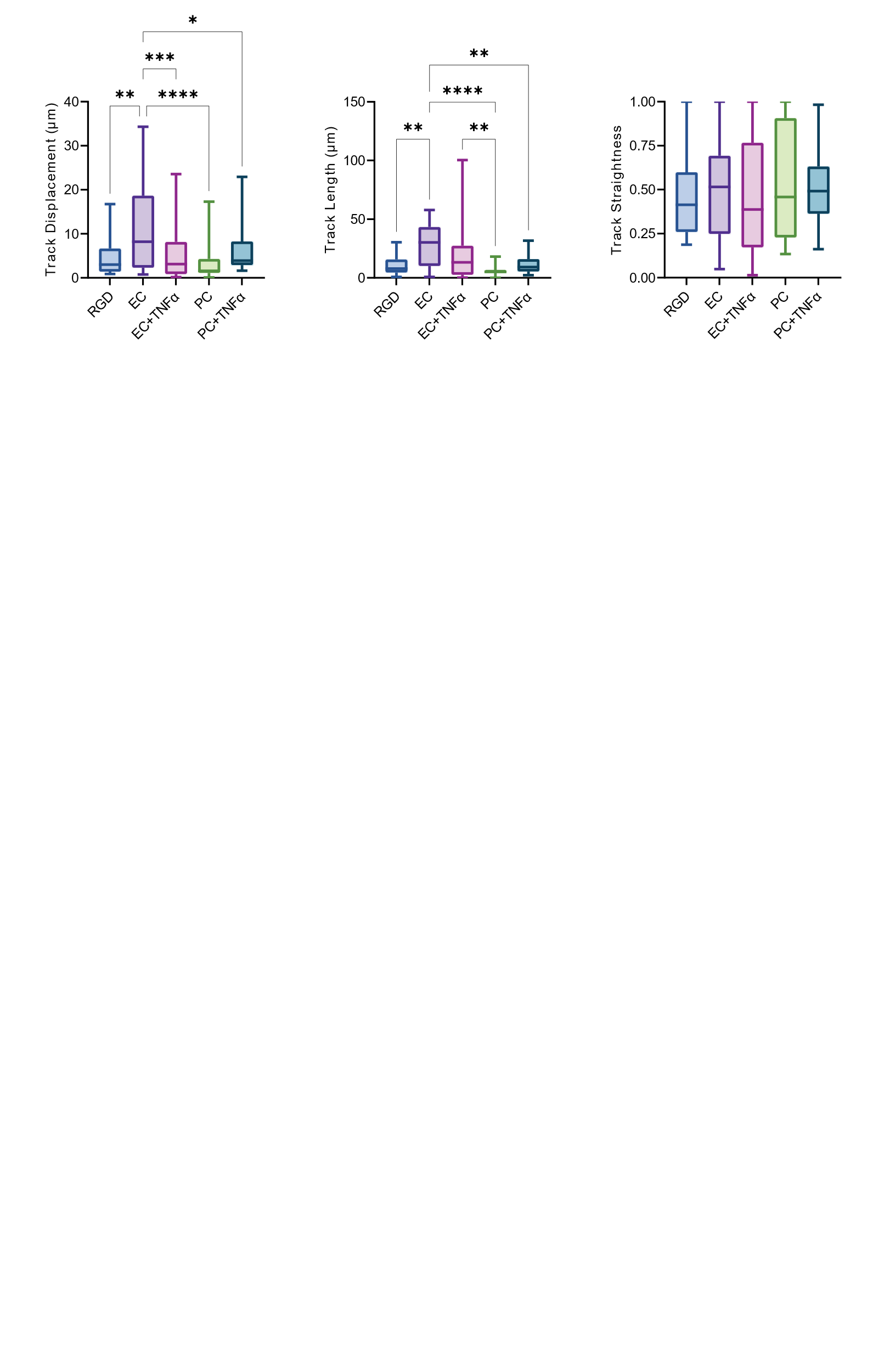


**Supplemental Figure 5. Additional Parameters Obtained from IMARIS Analysis of 4D Neutrophil Migration Data.** Box and whiskers plots illustrate three additional parameters of neutrophil migration: track displacement, length, and straightness. These metrics are compared across neutrophils seeded on a standalone fibrillar matrix versus those seeded on vascular cells, with further subgroup analysis based on the presence or absence of TNFα activation. One-way ANOVA was used to assess the significance of differences observed across conditions. Significance levels are denoted with asterisks representing p-values as follows: * (p ≤ 0.05), ** (p ≤ 0.01), *** (p ≤ 0.001), **** (p ≤ 0.0001).


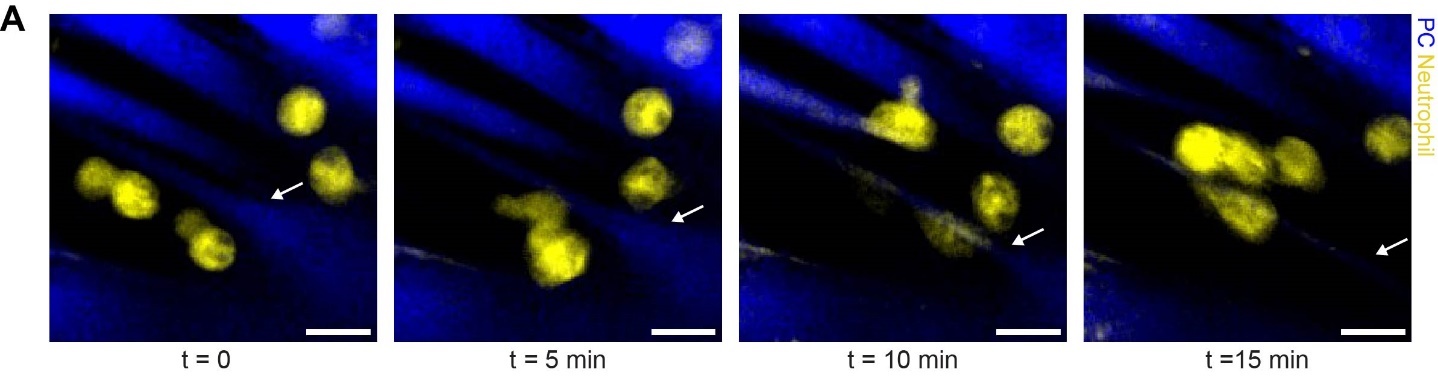


**Supplemental Figure 6. Preliminary observations of PC activated with TNF-α during interactions with neutrophils.** Confocal microscopy time-lapse imaging captures PC (blue) contractility in real-time as they interact with neutrophils (yellow). Scale bar: 10 µm.
